# Significance of Detection of the HER2 Gene and PD-1/PD-L1 in Gastric Cancer

**DOI:** 10.1155/2020/8678945

**Published:** 2020-10-13

**Authors:** Tian Yun, Sunan Wang, Bo Jiang, Changsong Wang, Nianlong Meng, Xutao Yuan, Yangkun Wang

**Affiliations:** ^1^People's Liberation Army Joint Logistic Support Force 989th Hospital, LuoYang, Henan 471031, China; ^2^ShenZhen Polytechnic, ShenZhen 518110, China; ^3^People's Liberation Army Joint Logistic Support Force 990th Hospital, ZhuMaDian, Henan 463000, China; ^4^Department of Pathology, ShenZhen Hospital, Southern Medical University, ShenZhen 518110, China

## Abstract

**Objective:**

To explore the relationship between the HER2 gene and PD-1/PD-L1 in gastric cancer and its significance.

**Methods:**

Immunohistochemistry (IHC) and fluorescence in situ hybridization (FISH) were used to detect HER2 protein expression, HER2 gene amplification, and PD-1/PD-L1 expression in 78 cases of gastric cancer.

**Results:**

The expression rate of HER2 protein was 43.6% (34/78), of which 19.4% (14/78) were HER2 3+, 14.1% (11/78) were HER2 2+, and 11.5% (9/78) were HER2 1+. The results showed that 19.2% (15/78) of samples had HER2 gene amplification, 3.8% (3/78) of samples had a HER2/CEP17 ratio <2.0, and 19.2% (15/78) of samples had HER2 gene amplificationf and HER2 copy/cell ≥6.0, as detected by FISH. The positive rate of PD-L1 was 38.5% (30/78) in gastric cancer cells and 50.0% (39/78) in interstitial lymphocytes. The expression of the HER2 gene, PD-L1, and PD-1 in gastric cancer was correlated with the stage and lymph node metastasis of gastric cancer (*P* < 0.05).

**Conclusions:**

The combined detection of the HER2 gene and PD-1/PD-L1 in gastric cancer provides an important reference index for the prognosis of gastric cancer and the benefit of targeted antitumor drugs.

## 1. Introduction

Although the incidence of gastric cancer (GC) has fallen significantly in the United States and across the world over the past few decades, it still remains the fifth most common malignancy and the third leading cause of cancer death [1, 2]. There are approximately 900,000 new cases of GC per year and 723,000 registered deaths worldwide. Most patients with gastric cancer have reached the middle and late stages at the time of diagnosis. Even if perioperative chemotherapy or adjuvant chemotherapy is used, the survival rate of these patients is still low [3, 4]. Therefore, the search for new therapeutic approaches, such as molecular targeted therapy, has become a hot topic in gastric cancer research. The human epidermal growth factor receptor type 2 (HER2, also referred to as HER2/neu) gene is located on chromosome 17q2l and encodes a transmembrane tyrosine kinase receptor with a relative molecular weight of 185000 Da. At present, the probes for the detection of HER2 gene status are mostly double probes containing the HER2 gene and the centromeric region of chromosome 17 (CEP17), where the gene is located. It was found that HER2 protein was expressed in breast cancer, ovarian cancer, gastric cancer, and other cancers [5–7]. The inducing factors of gastric cancer are mainly related to the activation of carcinogenic factors and the deactivation of tumor suppressor factors. Meanwhile, the tumor cells' immune escape is accompanied by the apoptosis reaction, in which the coregulatory system is involved.

At present, emerging immunotherapy against the programmed death-1 (PD-1)/PD-1 ligand (PD-L) pathway is attracting much attention. The immune escape mechanism of the PD-1/PD-L1 pathway is that the combination of PD-L1 on the surface of tumor cells and PD-1 on the surface of T lymphocytes inhibits the activity of T cells so that tumor cells escape from the attack of T lymphocytes [8–10]. PD-1/PD-L1 immunotherapy aims to treat many kinds of tumors by blocking the PD-1/PD-L1 signaling pathway and restoring the human autoimmune system. Monoclonal antibodies against blocking the PD-1/PD-L1 pathway have entered the clinical stage, and it has been indicated that the therapeutic effect of gastric cancer is significant [11, 12]. In this paper, immunohistochemistry and fluorescence in situ hybridization (FISH) were used to detect HER2 gene amplification and expression of PD-L1 and PD-1 in gastric cancer tissue and to explore the target and correlation of antitumor drug therapy for gastric cancer to provide help for the prognosis and targeted antitumor drug therapy of gastric cancer.

## 2. Materials and Methods

### 2.1. Gastric Cancer Tissue Specimen Selection

From November 2015 to February 2019, 78 cases of radical gastrectomy specimens were collected from the Department of Pathology of 989 Hospital, 990 Hospital, and Shenzhen Hospital of Southern Medical University. There were 12 cases of papillary adenocarcinoma, 23 cases of tubular adenocarcinoma, 11 cases of mucinous adenocarcinoma, 7 cases of poorly cohesive carcinoma, and 25 cases of mixed adenocarcinoma. The average age was 57.4 years.

All surgical specimens were processed by pathologists. Specimens were fixed within 30 minutes after surgery and fixed with 10% neutral buffered formalin (NBF) for 8–48 hours. The volume ratio of the fixative to tissue was 10 : 1. Four to six specimens were cut from the central area, and the surrounding area of the tumor (the proximal and distal margin of the tumor, the tumor, and the adjacent gastric mucosa were not included), one at the deepest infiltrating point and one at the closest serosa layer, and all lymph nodes and cancer nodes were cut in different areas. Hematoxylin-eosin (HE) staining, immunohistochemistry, and gene detection were performed.

### 2.2. Immunohistochemistry (IHC)

Monoclonal antibodies of PD-L1 (ab230369), PD1 (ab230369), and HER2 (3b5, ab16901) were bought from Abcam company. Using the Envision method, the operation steps were performed strictly in accordance with the product manual, PBS was used as the negative control instead of the primary antibody, and placental villi and lymph nodes were used as the positive controls for PD-L1 and PD-1, respectively. The ready-to-use kit and the primary antibody were purchased from Fuzhou MaiXin Company.

The percentage of tumor cells exhibiting cell-surface staining for PD-L1 was scored by two independent pathologists who were unaware of outcomes. First, the tumor cell area was determined under 4× low-power microscope; then, under 10–40× microscope, the nuclear staining site of PD-L1 and the percentage of PD-L1 positive tumor cells were identified. Tumor cells with ≥5% positive localization of PD-L1 in the cell membrane and/or cytoplasm were considered positive, and tumor stromal cells with ≥5% positive localization of PD-1 in the cell membrane and/or cytoplasm were considered positive [9, 13]. HER2 positive results were considered as follows: The positive localization was on the cell membrane. There was no staining on the cell membrane, and the result was 0; if tumor cells were faint in membrane staining, the result was 1+; if the basement membrane, side membrane, or integrity membrane of tumor cells had weak to moderate staining, the result was 2+; if there was strong positive staining on basement membrane, side membrane, or integrity membrane of tumor cells, the result was 3+. The positive staining area was evaluated as follows: if the membrane of tumor cells had no staining, the sample was considered negative; if ≥80% of cells were stained, the sample was considered extensive type; if 21%∼79% of cells were stained, the sample was considered partial type; and if ≤20% of cells were stained, the sample was considered focal type. Two pathologists were employed to read the film.

### 2.3. Fluorescence In Situ Hybridization

Paraffin Pretreatment Kit II (mainly including pretreatment solution and protease solution) and the Path Vysion TM HER2 Probe Kit were purchased from Vysis. The pretreatment procedure and FISH procedure of paraffin-embedded gastric cancer tissue sections were carried out in accordance with the literature [14, 15] and the instructions on the kit.

The gastric cancers with IHC grade HER 2+ results underwent FISH analysis. First, the positive area of gastric adenocarcinoma cells was confirmed on IHC, and then, the same field of view from IHC stain was assessed by FISH under a 10× objective lens, and the whole section was observed under 40× objective lens. More than 75% of the cancer cell nuclei had a hybridization signal, which was regarded as a satisfactory result; the lens was replaced with a 100 × objective lens to count at least 30 cancer cells with complete boundaries, isolated and nonoverlapping. The evaluation criteria for HER2 gene amplification were as follows: HER2/CEP17 ratio ≥2.0, and average HER2 copies/cells ≥4.0: FISH positive; HER2/CEP17 ratio <2.0, average HER2 copies/cells <4.0: FISH negative. If the ratio of HER2/CEP17 was less than 2.0 and the average copy number/cell of HER2 was more than 4.0 and less than 6.0, the signals in at least 20 nuclei were counted again. If the results changed, the two results were comprehensively judged and analyzed. In this group, if the ratio of HER2/CEP17 was less than 2.0 and the average copy number of HER2/cell was more than 6.0, the sample was judged as FISH positive (referred to as high multibody cell for short). If many HER2 signals were connected into clusters, they were directly judged as FISH positive. This group was divided into cluster amplification, large granular amplification, and dot amplification.

### 2.4. Statistical Analysis

SPSS 22.0 software was used for statistical analysis of all data. The *χ*2 test was used to analyze the relationship between the expression of HER2, PD-1, and PD-L1 and the clinicopathological characteristics of gastric cancer. The correlation between the two methods was analyzed by Pearson correlation analysis, and *P* < 0.05 was considered statistically significant.

## 3. Results

### 3.1. Relationship between HER2 Gene Amplification and the Protein Expression Rate

**Figure 1:**
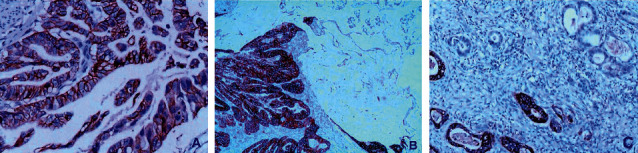
(a) Gastric papillary adenocarcinoma, HER2 positive 3+ extensive staining (X200); (b) Gastric mixed adenocarcinoma, mucinous adenocarcinoma, and papillary adenocarcinoma, HER2 positive 3+ partial staining (X40); (c) gastric tubular papillary adenocarcinoma, HER2 positive 3+ focal staining (X200), Envision method.

**Figure 2:**
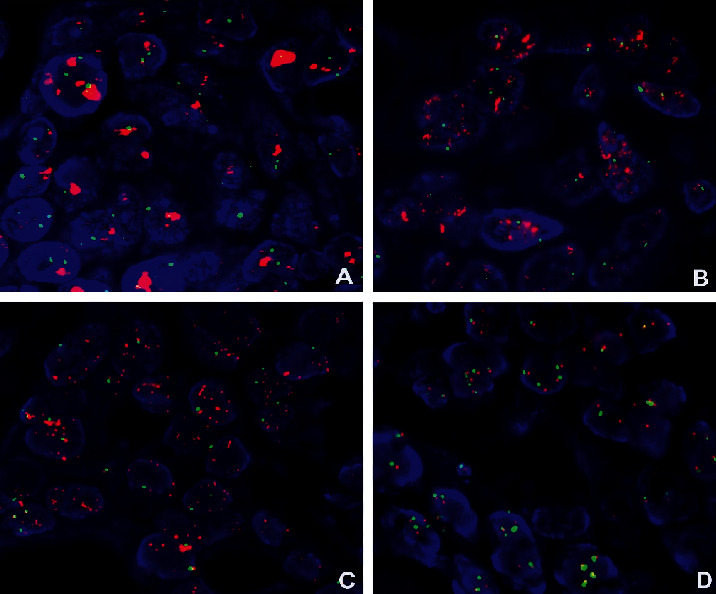
FISH method to detect HER2 gene amplification, red as probe signal, green as chromosome 17: (a) HER2 gene cluster amplification; (b) HER2 gene large particle amplification; (c) HER2 gene dot amplification; (d) HER2 gene high polymorph amplification.

HER2 protein was positively expressed in the cell membrane. In our search, the positive expression rate was 43.6% (34/78), in which the expression of HER2 protein 3+ accounted for 19.4% (14/78), including 8 cases of 3+ extensive staining (Figure 1(a)), 4 cases of partial staining (Figure 1(b)), and 2 cases of focal staining (Figure 1(c)); the expression of HER2 protein 2+ accounted for 14.1% (11/78), including 8 cases of 2+ extensive staining, 2 cases of partial staining, and 1 case of focal staining; the expression of HER2 protein 1+ accounted for 11.5% (9/78), including 7 cases of 1+ extensive type, 1 case of partial type, and 1 case of focal type. Forty-four cases were negative, accounting for 56.4% (44/78) of all cases. The amplification rate of the HER2 gene was 19.2% (15/78) by FISH technology, including 3 cases of HER2 gene cluster amplification (Figure 2(a)), 5 cases of large granule amplification (Figure 2(b)), 4 cases of dot amplification (Figure 2(c)), and 3 cases of high polymorph amplification (Figure 2(d)). The relationship between HER2 gene protein expression and HER2 gene amplification in gastric cancer is shown in Table 1.

### 3.2. Relationship of PD-1/PD-L1 Expression

**Figure 3:**
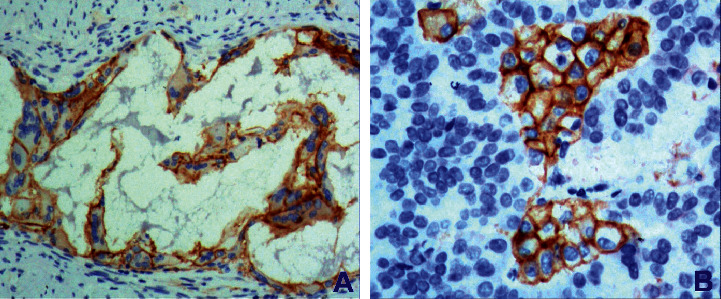
Gastric adenocarcinoma. (a) Gastric mucinous adenocarcinoma shows positive PD-L1 expression (X200). (b) Poorly differentiated gastric adenocarcinoma shows positive PD-L1 expression (X200), Envision method.

**Figure 4:**
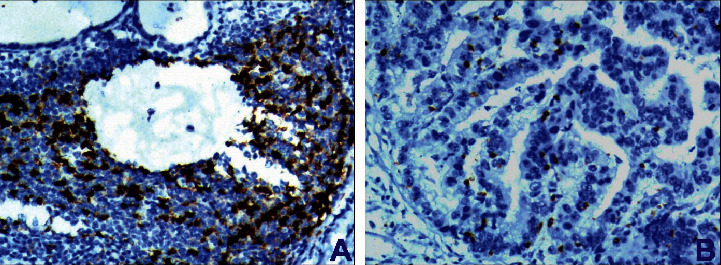
Gastric adenocarcinoma. (a) Interstitial lymphocytes of mucinous adenocarcinoma show positive expression of PD-1 (X200). (b) Interstitial lymphocytes of papillary gastric adenocarcinoma show negative expression of PD-1 (X200), Envision method.

The expression of PD-L1-positive cells in gastric cancer was multifocal and flaky (Figure 3); exfoliated cancer cells in the glandular cavity were expressed in different degrees. The total positive rate was 38.5% (30/78); PD-1 positive cells in tumor stroma were scattered or clumped: they were mainly located in the area of lymphocyte aggregation with focal distribution between adenocarcinoma cells and in the area of single lymphocyte between gastric adenocarcinoma cells (Figure 4). The total positive rate was 50.0% (39/78). The difference of PD-1/PD-L1 expression in tumor cells and tumor stroma was statistically significant (*P* < 0.05), and it was correlated with gastric cancer stage and lymph node metastasis (*P* < 0.05).

### 3.3. HER2 Gene Amplification and PD-1/PD-L1 Expression and Their Relationship with Clinicopathological Parameters

The amplification rate of the HER2 gene and the expression of PD-1/PD-L1 were not related to the sex and age of patients (*P* > 0.05); the amplification rate of the HER2 gene and the expression of PD-1/PD-L1 were related to the depth of invasion and lymph node metastasis of gastric cancer, with significant differences (*P* < 0.05), as shown in Table 2.

## 4. Discussion

The status of the HER2 gene determines the benefit of targeted treatment for gastric/GEJ adenocarcinoma. Therefore, accurate detection of the HER2 gene is very important for the selection of patients for targeted treatment. At present, detection of the HER2 gene in gastric/GEJ adenocarcinoma has become a routine project in the pathology department, and many countries have formulated HER2 detection guidelines [16, 17]. The detection of HER2 in gastric cancer is different from that in breast cancer, which has a wide range of morphological heterogeneities. The detection results are affected by many factors, as well as the quality control of the laboratory and the interpretation of staining results. Some studies indicated that the expression rate of HER2 protein was significantly different from 6% to 34% [18–21]. In our research, HER2 protein was detected by combining the positive intensity and the positive area, which was divided into extensive staining, partial staining, and focal staining. The total positive expression rate of HER2 protein was 43.6% (34/78), of which HER2 protein 3+ expression accounted for 19.4% (14/78), including 8 cases of 3+ extensive staining, 4 cases of partial staining, and 2 cases of focal staining; HER2 protein 2+ expression accounted for 14.1% (11/78), including 8 cases of 2+ extensive staining, 2 cases of partial staining, and 1 case of focal staining; HER2 protein 1+ expression accounted for 11.5% (9/78), including 7 cases of 1+ extensive staining, 1 case of partial staining, and 1 case of focal staining. Forty-four cases were negative, accounting for 56.4% (44/78) of all cases. The amplification rate of the HER2 gene was 19.2% (15/78), as detected by the FISH technique; amplifications included 3 cases of HER2 gene cluster amplification, 5 cases of large granule amplification, 4 cases of dot amplification, and 3 cases of high polymorph amplification. The results showed that HER2 amplification was correlated with gastric cancer stage and lymph node metastasis (*P* < 0.05).

There are many reasons for human tumors, which are closely related to the body's autoimmune function. Immunostimulatory molecules have become a hot spot in immunology in recent years. PD-1 and its ligand PD-L1 play an important role in tumor progression. PD-1 is a type I transmembrane protein composed of 288 amino acids, and PD-L1 is a type I transmembrane protein composed of 290 amino acids [11]. PD-1 binds to PD-L1 through the IGV domain of the extracellular domain. PD-1 is expressed on the surface of T cells, B cells, natural killer cells, and tumor infiltrating lymphocytes. PD-L1 is widely expressed in tumor cells. PD-L1 induces the binding of PD-1 on the surface of T-lymphocytes to transmit inhibitory signals to T cells, which inactivates T lymphocytes and suppresses the antitumor immune response [12]. Therefore, blocking the PD-1/PD-L1 pathway with drugs can enhance the function of T cells and cause tumor cell death, which will open a new window for tumor treatment. The expression of PD-1/PD-L1 in lung cancer, breast cancer, and malignant melanoma is higher than that in gastric cancer. The positive rate of PD-L1 expression in [13] tumor cells was 23.87%. The positive rate of PD-1 expression was 53.76%. PD-L1 was not expressed in normal gastric tissues but was detected in 42% of gastric cancer tissues. The positive rates of PD-L1 and PD-1 expression in gastric cancer cells were 38.5% and 50.0%, respectively. This study found that the expression of PD-1/PD-L1 was not related to the age of patients with gastric cancer but was related to the stage, lymph node metastasis, and prognosis of gastric cancer.

In recent years, it was reported that the status of the HER2 gene in gastric cancer is related to the expression of p53 protein and the number of Ki67-positive cells in cell proliferation [22, 23]. In this study, we found that HER2 gene status was related to gastric cancer stage, lymph node metastasis, and prognosis, which was consistent with the literature. The PD-1/PD-L1 pathway is an important mechanism of immunosuppression in the tumor microenvironment. Drugs are used to block this pathway and enhance immune function. By detecting the expression of PD-1/PD-L1 in gastric cancer, we can evaluate the prognosis of patients and provide some basis for immunotherapy of gastric cancer. It was also found that the detection of the HER2 gene and PD-1/PD-L1 in gastric cancer was related to gastric cancer stage and lymph node metastasis (*P* < 0.05).

In conclusion, HER2 protein is heterogeneous in gastric cancer, and the criterion and refinement of the HER2 test is the guarantee of correct medication guidance. This study emphasizes the intensity and scope of HER2 detection. HER2 protein expression can be divided into extensive staining, partial type, and focal staining. HER2 gene expression can be divided into cluster staining, large granule staining, dot staining, and high pleomorphism, which helps clinicians grasp the results of detection strategy, guide drug use, and predict prognosis. The PD-1/PD-L pathway is an important mechanism of immunosuppression in the tumor microenvironment. Drugs are used to block this pathway and enhance immune function. In this study, the HER2 gene and PD-1/PD-L1 were jointly detected in gastric cancer. All three genes were related to gastric cancer stage and lymph node metastasis (*P* < 0.05). This conclusion provides an important reference for the prognosis of gastric cancer and the benefit of targeted antitumor drugs.

## Figures and Tables

**Table 1:** Comparison of HER2 protein expression and HER2 gene amplification in 78 cases of gastric cancer.

	HER2 protein expression rate (%)	Amplification rate of HER2 gene (%)
−	44/78 (56.4)	0
+	9/78 (11.5)	0
++	11/78 (14.1)	1/11 (9.1)
+++	14/78 (19.4)	14/14 (100.0)
Total	34/78 (43.6)	15/78 (19.2)

**Table 2:** Relationship between HER2 gene amplification and PD-1/PD-L1 expression and clinicopathological parameters in 78 cases of gastric cancer.

Type	*n*	HER2 gene	*P* value	Expression of PD-L1 in tumor cells	*P* value	Expression of PD-1 in tumor stromal lymphocytes	*P* value
		Positive, negative		Positive, negative		Positive, negative	
Sex							
Male	53	11 (26.4), 42 (79.2)	0.619	22 (41.5), 32 (60.4)	0.516	29 (54.7), 24 (45.43)	0.377
Female	25	4 (16.0), 21 (84.0)		8 (32.0), 17 (68.0)		11 (44.0), 14 (56.0)	
Age							
≤60	31	6 (19.4), 25 (80.0)	0.982	9 (29.0), 22 (70.8)	0.164	13 (41.9), 18 (58.1)	0.247
>60	47	9 (19.1), 38 (80.9)		21 (44.7), 26 (55.3)		26 (55.3), 21 (44.7)	
Histological classification							
Papillary adenocarcinoma	12	3 (25.0), 9 (75.0)	0.924	5 (41.6), 7 (58.3)	0.991	6 (50.0), 6 (50.0)	0.977
Tubular adenocarcinoma	23	5 (21.7), 18 (78.3)		9 (39.1), 14 (60.9)		11 (47.8), 12 (52.2)	
Mucinous adenocarcinoma	11	1 (9.1), 10 (90.0)		4 (36.4), 7 (63.6)		5 (45.5), 6 (54.5)	
Low adhesion cancer	7	1 (14.3), 6 (85.7)		2 (28.6), 5 (71.4)		3 (42.9), 4 (57.1)	
Mixed adenocarcinoma	25	5 (20.0), 20 (80.0)		10 (40.0), 15 (60.0)		14 (56.0), 11 (44.0)	
Stage							
T1/T2	30	2 (6.7), 28 (93.3)	0.026	8 (26.7), 22 (73.3)	0.129	9 (30.0), 21 (70.0)	0.005
T3/T4	48	10 (20.8), 38 (79.2)		21 (43.8), 27 (56.3)		30 (62.5), 21 (37.5)	
Lymph node metastasis							
With	56	12 (21.4), 44 (78.6)	0.787	26 (43.4), 30 (53.6)	0.021	32 (58.2), 23 (41.8)	0.037
Without	22	3 (13.6), 9 (40.9)		4 (18.2), 18 (81.8)		7 (31.8), 15 (68.2)	

## Data Availability

All the data generated or analyzed during this study are included within this article.
